# Effect of Zinc Oxide Nanoparticles as Feed Additive on Blood Indices, Physiological, Immunological Responses, and Histological Changes in Broiler Chicks

**DOI:** 10.1007/s12011-023-03820-y

**Published:** 2023-09-04

**Authors:** Mahmoud H. Hatab, Aml M. M. Badran, Mahmoud A. Elaroussi, Eman Rashad, Adel M. Abu Taleb, Abdelmotaleb A. Elokil

**Affiliations:** 1 Biological Application Department, Nuclear Research Center, Egyptian Atomic Energy Authority, P.O. Box 13759, Cairo, Egypt; 2 Poultry Breeding Department, Animal Production Research Institute, Agriculture Research Center, Ministry of Agriculture, Dokki, Giza, Egypt; 3 Cytology and Histology Department, Faculty of Veterinary Medicine, Cairo University, Giza, Egypt; 4 Animal Production Department, Faculty of Agriculture, Benha University, Moshtohor, Egypt

**Keywords:** Zinc oxide nanoparticles, Blood indices, Antioxidants, Immune response, Broilers

## Abstract

A feeding trial of 5-week duration was performed to assess the response of broiler chicks to dietary supplementation with different doses of myco-fabricated zinc oxide nanoparticles (ZONPs) on blood indices, physiological, immunological response, antioxidant status, intestinal microbial count, and histological changes in immune organs. A total of 162 3-day-old Ross 308 broiler chicks were weighed individually and distributed equally into 3 dietary treatments with 6 replicate of 9 chicks in each in a completely randomized design. Chicks were fed ad libitum a basal ration prepared as starter, grower, and finisher supplemented with 0 (T1, control), 40 (T2), and 60 (T3) mg zinc oxide nanoparticles (ZONPs)/kg feed. Results showed that supplementing with ZONPs at both studied levels increased the relative weights of the spleen, bursa, thymus, and liver and decreased the relative weight of the kidney, gizzard, and intestine. A significant increase in the concentrations of hemoglobin (Hb), hematocrit (PCV%), red and white blood cell counts, total protein (TP), globulin (GLOB), aspartate transferase (AST), alanine transferase (ALT), alkaline phosphatase (ALP), superoxide dismutase (SOD), glutathione peroxidase (GPx), and total antioxidant capacity (TAC) and a significant decrease in malonaldehyde (MDA), uric acid, and creatinine concentration were observed. Furthermore, all immunological organs showed histological alteration and increased both types of immunity in ZONPs groups with more pronounced effects in the T2 group.

## Introduction

The National Research Council’s (NRC) recommendations of 40 ppm of zinc [1] have not been found enough to support the optimum development potential of the current high-yield commercial strains [2, 3]. Traditionally, inorganic zinc has been added to commercial diets as a classical form of supplementation [4, 5]. However, a higher dose of zinc (110 mg/kg feed) may be antagonistic and affect the bioavailability of other minerals and vitamins, which results in a low rate of absorption of inorganic zinc [6, 7].

Recently, Zn in nano form (ZONPs) have attracted a great attention as alternative feed supplements to both organic and inorganic Zn sources with regard to absorption, bioavailability, stability, inexpensive, and efficacy at the same or even low doses and seem also to be less toxic [8, 9]. In addition, ZONPs have been demonstrated to exert more positive effects, producing better results, due to their novel properties, such as smaller size, increased surface area, a high number of surface active centers, stronger adsorbing quality, catalytic efficiency, increased bioavailability, absorbability, and exerting a superior efficacy [10, 11].

Biological synthesis (green methods) using microorganisms is the latest trend in the nanotechnology and has emerged as an alternative to chemical and physical methods [12–14]. Biogenic synthesis nanoparticles have several benefits, as it being easier, simpler, and does not involve any hazardous chemicals, affordable, safe, clean, controlled NPs size, and eco-friendly, as well as achieving the desired with high-yield and high-value NPs compounds [12, 15, 16].

The objective of the current study was to evaluate the impact of using biosynthesis ZONPs as feed additives in broiler diets on some hematological and biochemical changes, immune competence, antioxidant status, antibacterial effect, and histological changes in immune organs.

## Material and Methods

### Birds, Husbandry, Management, and Experimental Design

This experimental study was conducted at the experimental poultry farm of the poultry physiology and production unit, Biological Application Department, Radioisotopes Applications Division, Nuclear Research Center, Egyptian Atomic Energy Authority, Egypt.

A total of one hundred and eighty 1-day-old broiler chicks (Ross308) were housed in batteries and fed basal diets without receiving any treatments until they were 3 days old as quarantine and adaptation period to make birds adapt to cage rearing and ensure they were free of pathogens and other diseases.

At 3 days of age (experimental initiation), all chicks were weighed individually, and the upper and lower body weights of average were excluded. The remaining 162 chicks were utilized from the 3rd day to the 5 weeks of age (38 days) (experimental termination). According to the dosage of supplemented zinc oxide nanoparticles (ZONPs), the chicks were divided equally by weight and number in a completely randomized design into 3 dietary treatments. Each treatment was further equally replicated 6 times with 9 birds per replicate **(**54 birds/treatment), and each replicate was housed separately by placing cages in different locations within the same room.

The dietary treatment groups were arranged as group one receiving the basal diet without ZONPs supplementation and functioned as the control group (T1), while the other two treatment groups received the basal diet supplemented with ZONPs at 40 and 60 mg/kg diet (T2 and T3, respectively). The chicks were reared in galvanized stainless steel clean batteries cages in the same room in an open-sided house that had previously been fumigated with formalin and potassium permanganate. The house is environmentally controlled under the same identical management and standard hygienic conditions.

The batteries were supplied by suitable feeder and fresh drinking water via stainless steel drip nipple whose height was continually adjusted to the age of the birds, allowing the birds to have unlimited access to feed and water. The room temperature was adjusted according to the age of the birds, starting with 34 °C for the first 3 days and decreasing gradually after that by 2 °C weekly until reaching 24 °C at the end of the fifth week. Artificial lighting was used to provide a lighting program of 24 h of light per day for the first 3 days and a 23-h light:1-h dark cycle per day until the experiment’s termination. The birds were under clinical observation, with special attention paid to the activity of the birds, their appetite, respiratory symptoms, and the occurrence of digestive disorders. The birds were vaccinated against Marek’s disease, Newcastle disease, and infectious bursal disease during the experiment as per schedule.

### Experimental Diet

The experimental diets were formulated to maintain the nutritional requirement of broilers and varied between groups in ZONPs supplementation. Chicks were fed according to 3 feeding periods: the starter period (0–12 days), the grower period (13–28 days), and the finisher period (28–35 days). All experimental diets within a period had the same chemical composition and were formulated of corn-soybean meal basal diet, provided to fed in mash form. The diets were formulated based on the nutrient composition of the feedstuffs to cover all nutrient needs of Ross 308 chicks according to the guidelines by the Ross nutrition specifications for a target live weight of 1.7–2.4 kg [17], except for Zn groups.

The basal diets were formulated using Zn-free mineral premix. Before the trial, 5 samples of each of the starter, grower, and finisher diets were analyzed for their zinc content by inductively coupled plasma optical emission spectrometer (Leeman Prodigy High Dispersion ICP-OES, USA) according to the method described by Sunder et al. [18] and were found to contain 23.55, 22.1, and 20.17 mg/kg of Zn from raw materials, respectively. The required amount of ZONPs in each treatment level was added to a premix and then mixed well with each aliquot of the basal diet. The experimental diet composition, nutritional value, and chemical analysis are presented in Table 1.

**Table 1:** Formulation, ingredient composition, and chemical analysis for starter, grower, and finisher diets

Ingredient composition (kg)	Starter	Grower	Finisher
Yellow corn	54	56.2	61.85
Soybean meal 44%	34.5	29	25
Corn gluten meal (60%)	5.5	6.35	5
Vitamin and mineral premix	0.3	0.3	0.3
Dicalcium phosphate	2	1.5	1.3
Sodium chloride	0.3	0.3	0.3
Vegetable oil	1.8	4.2	4.5
Limestone	1.1	1.6	1.3
dl-Methionine	0.2	0.3	0.3
l-Lysin	0.29	0.25	0.17
Calculated chemical analysis			
Crude protein %	23	21.5	19.4
Metabolizable energy	3001	3159	3237
Calcium	0.99	1.03	0.87
Lysine	1.43	1.25	1.06
Available phosphorus	0.51	0.47	0.45
Methionine	0.59	0.52	0.46
Methionine + cysteine %	0.93	0.81	0.72

Vitamin-mineral premix provided per kg of diet: vitamin A, 12,000 IU; vitamin D3, 4000 IU; vitamin E, 55 IU; vitamin K3, 3 mg; biotin, 0.18 mg; vitamin B1, 2.3 mg; vitamin B2, 6.5 mg; pantothenic acid, 20 mg; vitamin B6, 4mg; niacin, 60 mg; vitamin B12, 0.017 mg; choline, 150 mg; folic acid, 1.9 mg. Mn, 120 mg; Fe, 20 mg; Cu, 15 mg; I, 0.20 mg; Se, 0.3 mg; Zn, 0 mg

### Zinc Oxide Nanoparticles (ZONPs) Synthesis, Preparation, and Purification

In the present study, ZONPs were synthesized by the extracellular myco-synthesis technique using the cell-free culture filtrate of the fungus *Alternaria tenuissima* AUMC1062 according to the method described in detail in our previous report [12]. Fungal spores from 7-day-old *A. tenuissima* culture were harvested and exposed to 500 Gy of gamma rays, the optimal exposure level to maximize ZONPs synthesis [19]**.** Irradiation process was carried out at the Nuclear Research Center (Cairo, Egypt) using ^60^Co Gamma chamber, MC20, Russia, with an average dose rate of 605.726 Gy h^−1^ at the time of the experiment. Characterization of the biogenic ZONPs was accomplished by various techniques. ZONPs solution was analyzed on UV–Vis spectrophotometer (UV-3101PC; Shimadzu, Kyoto, Japan). X-ray diffraction (XRD) was used to identify the crystalline nature of ZONPs. The shape and size of ZONPs were measured by transmission electron microscopy (TEM). Dynamic light scattering (DLS) and zeta potential analyses (Zetasizer Nano ZS, Malvern Instruments, Worcestershire, UK) were carried out to estimate the distribution of different size particles dispersed in ZONPs solution and its stability according to previous reports [20].

### Sampling and Measurements

#### Organs Index, Blood Sampling, Hematological, and Biochemical Analysis

At the end of experiment, 3 birds from each replicate (18 birds/group) with the average body weight near to the mean body weight of each group were chosen, fasted for 6 h with free access to water, then weighed individually and slaughtered to full bleeding. Some organs (liver, kidney, gizzard, and intestine) and immune organs (spleen, bursa, and thymus) were extracted, blotted to dry, and then weighed individually to calculate their relative weights to fasted live body weight (g/100 g). Furthermore, a histological study was also carried out to assess the histological changes in the spleen, bursa, and thymus.

Blood samples were collected at the time of slaughter from the same 18 birds using sterile clean tubes per bird. The first blood sample was collected into heparinized tubes as an anticoagulant and used to determine hemoglobin concentration (Hb%), packed cell volume percentage (PCV%) [21], red blood cells (RBCs), and white blood cells (WBCs) counts [22], while the second blood sample was collected into a sterile clean centrifuged tube without anticoagulant, left to coagulate at room temperature, and immediately centrifuged at 4000 rpm for 10 min to obtain serum. The obtained serum was transferred to dry sterile screw-capped tubes (Eppendorf) and stored in a deep freezer (− 20 °C) to perform subsequent biochemical test.

Serum content of total proteins (TP) and albumin (ALb) was measured spectrophotometrically, and serum globulin was calculated by subtracting the obtained serum albumin value from the corresponding total protein value for each sample, whereas the A/G ratio was calculated according to results of albumin and globulin. Serum enzyme activity of alanine aminotransferase (ALT), aspartate aminotransferase (AST), and alkaline phosphates (ALP) as indicators of liver function and uric acid and creatinine as indicators for kidney function were analyzed using a spectrophotometer (SHIMADZU UV 1601) using specific commercial kits (Bio Med, Hannover, Germany) according to the manufacturer’s instruction. Also, serum concentrations of superoxide dismutase (SOD), glutathione peroxidase (GPx), malondialdehyde (MDA), and the total antioxidant capacity (TAC) were determined spectrophotometrically using the commercially available respective detection kits (Bio-diagnostics) according to the procedures provided by the manufacturers.

#### Immunological Responses

The immune system function was estimated by both humoral and cell-mediated immune response as follows:

- Humoral immune response

Humoral immune response was determined by two tests, namely hemagglutination test (HA) for the detection of antibody produced against sheep red blood cells (SRBCs) inoculation and hemagglutination inhibition test (HI) for the detection of antibody produced against Newcastle disease vaccination.

- Determination of humoral immune response by HA test against sheep red blood cells (SRBC)

SRBCs, as nonpathogenic antigen, were employed for evaluating the primary and secondary immune responses in broiler by HA test.

Three birds were chosen from each replication that were near to the average body weight of each treatment (18 birds from each group), marked with dye. At 15 days, each bird received a first intravenous injection of 0.5 mL of 5% fresh SRBC suspension in either the right or left wing vein (first injection to determine the primary immune response). Ten days after the first injection, the birds received a booster injection with the same dosage of SRBC (second injection to determine the secondary immune response). After each SRBC injection, blood samples were collected from the bird’s wing vein at 3, 7, and 10 days later [23]. After allowing blood to coagulate, serum was immediately separated using centrifugation. Serum samples were subsequently analyzed for antibodies produced against SRBCs by hemagglutination test (HA) according to Peterson et al. [24]**.** Each bird’s serum sample was titrated individually in microtiter plates, and the plates were read under the bright light. The antibody titers were expressed as the log base^2^ (Log^2^) of the reciprocal of the highest dilution giving a visible agglutination [25].

- Determination of humoral immune response by HI test against Newcastle disease virus

At 18 days of age, all chicks were vaccinated against Newcastle disease virus with Live LaSota strain vaccine (Volvac) via eye droplets as the second vaccination after the first vaccination that was done at 8 days of age with Hitchner B1. After vaccination with the LaSota strain, 18 birds from each group (3 birds/replicate) were chosen. Blood samples were drawn from the brachial vein of chosen chicks after 3, 7, and 10 days from vaccination, and the serum was obtained by centrifuging the blood. The presence of antibodies generated against Newcastle in serum samples was assessed by hemagglutination inhibition (HI) test according to Poorghasemi et al. [26]. The virus-antibody titer was determined as the reciprocal of the highest dilution of the tested serum that completely inhibited the activity of the virus HA (button-like pattern). Antibody titers were obtained by transforming the values of the last dilutions which produced total inhibition of hemagglutination into values of antibody titers and expressed as the logarithm to the base^2^ (Log^2^).

- Cell-mediated immune response

Cell-mediated immune response was evaluated in vivo by cutaneous basophil hypersensitivity (CBH) test via the response of the wattles to injection with the mitogen phytohaemagglutinin type P (PHA-P) (PECTIN FROM PHASEOLUS GARIS, Sigma, code number L 8754, lot 10k7606) according to the method of Corrier and DeLoach [27], where 15 mg of the lyophilized powder (PHA-P) was diluted in 15 mL of physiological saline solution (PS) in order to obtain a dose of 100 µg/0.1 mL per bird.

At 32 days of birds’ age, 18 birds (3 birds/replicate) from each group were selected and housed in separate cages. Each bird received an intradermal injection in the right wattle with 0.1 mL of a solution of PHA-P in sterile physiological saline (1 mg/mL). The left wattles will serve as control and were injected intradermally with 0.1 mL of sterile saline solution. The thickness of the two wattles was measured by a digital skinfold caliper immediately before and 24 h after injection. Relative response (R.R) was calculated by dividing the thickness obtained for the wattle-injected PHA on the thickness obtained for the wattle-injected saline (right/left), 24 h post-injection for individual bird. Also, the wattle index (W.I) was calculated by subtracting the thickness of the two wattles before injection from the thickness 24 h after injection for individual bird and was expressed as millimeter. A wattle swelling which exceeded 0.5 mm was considered a positive response since the saline-injected wattle never exceeded this measurement at 24 h. Results were expressed as the ratio of the thickness of the wattle injected with the antigen to that PBS-injected control wattle.

#### ***Intestinal Microbial Count (Enumeration of Bacteria Populations in Ileum and Cecum (CFU/g)*** × ***10***.^***6***^***)***

The fresh samples from both ileum and cecum from the same birds slaughtered of the three treatment groups at the end of experiment were immediately collected using a sterile sharp blade under aseptic conditions. Collected samples were immediately put in sterile glass bags and placed on ice, until they were transported to the laboratory for enumeration of microbial populations in these 2 sections. The microbial populations of *Lactobacilli*, *Bifidobacterium*, *Escherichia coli*, and *Salmonella* were enumerated in each part using commercial-specific agar media for each kind of bacteria [28]. Briefly, 1 cm of each sample containing feces was transferred into a sterile Erlenmeyer flask containing sterile saline peptone water (1 g/L peptone and 8.5 g/L NaCl) and thoroughly homogenized by vortexing for 10 min. After which, 1 mL of each was added to 9 mL sterile saline solution and vortexed for another 10 min (first dilution) and repeated for a second time to make serial dilutions up to 10^6^. Inoculate 0.1 mL from each dilution in sterile petri dishes contains differential sterilized medium (specific for each kind of bacteria) as MRS agar (De Man, Rogosa, and Sharpe agar), EMB (Eosin methylene blue agar), SSA (Salmonella Shigella agar), and BSM agar (Bifidobacterium Selective Media) for *Lactobacillus*, *E. coli*, *Salmonella*, and *Bifidobacterium* respectively. The dishes were incubated for 48 h at 37 °C for *Lactobacillus* and *Bifidobacterium* whereas *E. coli* and *Salmonella* agar plates were incubated for 24 h at 37 °C. After incubation, the colonies on each plate were enumerated immediately. The bacterial colonies’ numbers were counted and the results were converted to log number to be ready for the statistical analysis. The average number of bacterial counts was calculated and expressed as colony-forming unit (CFU)/g [29].

Count(cfu) = (number of colonies/inoculum taken) * dilution.

#### Histological Examination

The spleen, bursa, and thymus gland from the same 18 birds slaughtered of each treatment group were collected for histological studies to evaluate the presence of histopathological lesions. The procedure for histological preparation is as described by Bancroft and Layton [30].

### Statistical Analysis

The obtained data were statistically analyzed using one-way analysis of variance (ANOVA) as a completely randomized design using the general linear models (GLM) procedure under statistical analysis system software [31]. The data are presented as means ± standard error (SEM). Significant differences between treatment means were compared by Duncan’s multiple-range test [32] and were considered to be statistically significant at *P* < 0.05.

## Results

Firstly, the overall health of every bird groups was excellent, and no respiratory or gastrointestinal syndromes or deaths were recorded during the entire study period.

### Characterization of Zinc Oxide Nanoparticles

The cell-free filtrate of *Alternaria tenuissima* culture was added to the colorless bulk aqueous zinc sulfate solution. After 20 min of incubation, a white precipitate was observed, indicating the complete reduction of zinc sulfate to ZONPs. The synthesis of ZONPs from zinc sulfate was verified using UV–Vis spectrophotometric analysis. The produced ZONPs showed an absorption peak at 369 nm in the UV–Vis spectra. The results showed that the produced ZONPs have a hexagonal type of crystal structure. The biogenic synthesis ZONPs exhibited a single-phase crystalline structure with a spherical shape and on the nanoscale, according to the X-ray diffraction (XRD) results. ZONPs were found to be mono-dispersed by dynamic light scattering (DLS) examination, and a polydispersity index with a value of 0.308 was obtained. The reported average zeta potential value of − 23.81 mV proved the great stability of ZONPs. According to transmission electron microscopy (TEM) findings, the generated ZONPs nanoparticles had an average diameter and particle size of about 15.51 nm.

### Relative Organ Weights

Table 2 presents the changes that occurred in the weights of both digestive and immune organs relative to body weights in their response to dietary ZONPs. Statistical analysis (*P* < 0.05) indicated that both studied doses of ZONPs induced a significant decrease in the relative kidney, gizzard, and intestine weights and a significant increase in relative lymphoid organ weights (spleen, bursa, and thymus) as compared with control. On the other hand, liver weight was increased at both ZONPs-supplemented groups than control in a dose-dependent manner, but this increase was only significant with increasing ZONPs dose (T3).

**Table 2:** Effects of ZONPs supplementation on relative digestive and immune organs weights of broiler chicks (percent of live body weight)

Groups	Spleen %	Bursa %	Thymus %	Liver %	Kidney %	Gizzard %	Intestine %
T1	0.0894^b^ ± 0.006	0.191^b^ ± 0.011	0.384^b^ ± 0.015	2.032^b^ ± 0.015	0.711^a^ ± 0.03	2.715^a^ ± 0.152	5.852^a^ ± 0.23
T2	0.127^a^ ± 0.012	0.249^a^ ± 0.011	0.442^a^ ± 0.019	2.074^b^ ± 0.046	0.487^b^ ± 0.022	2.334^b^ ± 0.122	4.926^b^ ± 0.28
T3	0.113^a^ ± 0.008	0.223^a^ ± 0.009	0.413^a^ ± 0.016	2.447^a^ ± 0.011	0.501^b^ ± 0.025	2.451^b^ ± 0.13	5.014^b^ ± 0.27

Values are means ± SEM

^a,b^The same column with different superscripts are significantly different (*P* < 0.05)

### Hematological Parameters

The influences of dietary supplementation with ZONPs on some hematological measurements in broilers are summarized in Table 3. Results obtained revealed that broiler chicks fed a supplemented diet with 40 ppm ZONPs recorded significantly the highest effect on all hematological variables (Hb%, PCV%, RBCs, and WBCs counts) compared to the control group. The birds given 60 ppm ZONPs were shown a significant increase in PCV and WBCs with a nonsignificant increase in Hb and RBCs count when compared with control birds. No significant differences were observed between the two doses of ZONPs.

**Table 3:** Effects of ZONPs supplementation on hematological parameters of broiler chicks

Groups	Hb%	PCV%	RBCs (10^3^ × mm^3^)	WBCs (10^6^ × mm^3^)
T1	11.66^b^ ± 0.36	36.0^b^ ± 1.24	3.695^b^ ± 0.23	14.42^b^ ± 0.68
T2	12.53^a^ ± 0.29	39.3^a^ ± 1.18	4.092^a^ ± 0.19	16.43^a^ ± 0.51
T3	12.22^ab^ ± 0.31	38.6^a^ ± 1.08	3.923^ab^ ± 0.17	15.8^a^ ± 0.58

Values are means ± SEM

^a,b^The same column with different superscripts are significantly different (*P* < 0.05)

*Hb* hemoglobin, *PCV* packed cell volume (hematocrit), *RBCs* red blood cells count, *WBCs* white blood cell count

### Biochemical Parameters

Table 4 presents information on blood biochemical estimates in broilers as a results of ZONPs supplementation. Results revealed that birds fed a supplemented diet with ZONPs had higher serum total proteins (TP) and globulins (GLOB) at both levels than controls, but this difference was only significant at 40 ppm. A highest content of serum aspartate aminotransferase (AST) and alanine aminotransferase (ALT) was observed in ZONPs-supplemented groups and became statistically significant only when the inclusion level was increased to 60 ppm. Both of the tested doses of ZONPs increased alkaline phosphatase (ALP) significantly. Conversely, serum uric acid and creatinine (creat) concentrations were decreased in tested birds in a dose-dependent manner as compared with control birds.

**Table 4:** Effects of ZONPs supplementation on biochemical measurements of broiler chicks

Groups	T.P (g/dL)	ALB (g/dL)	GLOB (g/dL)	A/G ratio	ALT (U/L)	AST (U/L)	ALP (u/L)	Uric acid (mg/dL)	Creat (mg/dL)
T1	3.933^b^ ± 0.15	2.083^a^ ± 0.062	1.850^b^ ± 0.22	1.13^a^ ± 0.05	29.364^b^ ± 1.23	24.22^b^ ± 1.25	165.41^b^ ± 1.52	7.05^a^ ± 0.25	0.772^a^ ± 0.026
T2	4.333^a^ ± 0.096	2.107^a^ ± 0.046	2.226^a^ ± 0.099	0.95^a^ ± 0.02	30.113^b^ ± 1.55	24.55^b^ ± 1.31	168.2^a^ ± 1.44	6.88^a^ ± 0.41	0.711^b^ ± 0.03
T3	4.067^ab^ ± 0.12	2.078^a^ ± 0.061	1.989^ab^ ± 0.18	1.045^a^ ± 0.03	32.106^a^ ± 1.14	25.142^a^ ± 1.35	174.6^a^ ± 1.57	5.79^b^ ± 0.27	0.688^b^ ± 0.027

Values are means ± SEM

^a,b^The same column with different superscripts are significantly different (*P* < 0.05)

*TP* total proteins, *ALB* albumin, *GLOB* globulins, *ALT* alanine transferase, *AST* aspartate transferase, *ALP* alkaline phosphatase, *Creat* creatinine

### Serum Antioxidant Capacity

The effects of dietary ZONPs supplementation on the serum antioxidant enzyme profile of broilers are presented in Table 5. Serum content of superoxide dismutase (SOD), Glutathione peroxidase (GPx), and total antioxidant capacity (TAC) was increased significantly in ZONPs groups as compared with control birds. In contrast, serum malonaldehyde (MDA) concentration was significantly decreased in a dose-dependent way in both treated groups with ZONPs when compared with control one.

**Table 5:** Effects of dietary ZONPs supplementation on serum oxidative stress biomarkers of broilers

Groups	MDA (nmol/mL)	SOD (U/mL)	GPX (µ/mL)	TAC (mM/L)
T1	4.359^a^ ± 0.15	127.6^b^ ± 1.67	15.14^b^ ± 1.55	0.617^b^ ± 0.05
T2	3.543^b^ ± 0.17	133.7^a^ ± 1.51	18.23^a^ ± 1.14	1.261^a^ ± 0.02
T3	3.719^b^ ± 0.17	134.3^a^ ± 1.55	18.02^a^ ± 1.23	0.992^a^ ± 0.02

Values are means ± SEM

^a,b^The same column with different superscripts are significantly different (*P* < 0.05)

*MDA* malondialdehyde, *SOD* superoxide dismutase, *GPX* glutathione peroxidase, *TAC* total antioxidant capacity

### Immunological Parameters

#### Humoral Immune Response

The effects of dietary ZONP inclusion on the antibody titers against SRBC and NDV a measure of humoral immune response are presented in Table 6. The results clarified that the control group had inferiority in antibody titers immunization days. It was evident that supplemented broiler diets with ZONPs resulted in an increase in the antibodies produced against NDV and SRBC antigens at all test times as compared to the amount of antibody produced in controls.

**Table 6:** Effects of dietary ZONPs supplementation on antibody titer against SRBC and NDV after 3, 7, and 10 days post-immunization of broiler chicks

Groups	HA titer against SRBCs						HI titer against NDV		
	Days post-immunization (primary immune response)			Days post-immunization (secondary immune response)			Days post-immunization		
	3	7	10	3	7	10	3	7	10
T1	2.567^a^ ± 0. 48	4.414^b^ ± 0.72	3.725^b^ ± 0.5	3.20^b^ ± 0.40	5.80^b^ ± 0.51	4.40^b^ ± 0.68	3.60^ab^ ± 0.49	6.00^c^ ± 0.60	5.00^b^ ± 0.51
T2	2.755^a^ ± 0.62	5.512^a^ ± 0.54	5.147^a^ ± 0.44	4.60^a^ ± 0.40	7.00^a^ ± 0.55	5.20^a^ ± 0.66	4.20^a^ ± 0.37	7.80^a^ ± 0.37	6.40^a^ ± 0.53
T3	2.701^a^ ± 0.64	5.355^a^ ± 0.61	4.868^a^ ± 0.52	4.20^a^ ± 0.37	6.60^a^ ± 0.51	5.00^ab^ ± 0.71	4.00^a^ ± 0.45	7.20^b^ ± 0.49	6.00^a^ ± 0.48

Values are expressed as mean ± SEM based on 18 birds taken at random from each group

^a,b,c^The same column with different superscripts are significantly different (*P* < 0.05)

*HA* and *HI* are hemagglutination and hemagglutination inhibition tests to determine the antibody titer against SRBCs and NDV respectively

#### Cell-Mediated Immunity

Results in Table 7 refer to the effects of ZONPs supplementation on the cell-mediated immune response to wattle injections with PHA-P. It could be noticed that the left control wattle in all tested chickens of the three examined groups (which was injected with 0.1 mL of sterile saline solution) had the same thickness 24 h post-injection as that before injection. Concerning the right wattle (which was injected with 0.1 mL of PHA solution), statistical analysis revealed that both groups fed dietary ZONPs (T2 and T3) supplementation respond better as indicated by a significant increase in the relative response (R.R) and wattle index (W.I) as compared to control birds. The highest relative response and wattle index were recorded among the T2 birds which measured at 1.59 and 0.907 mm thickness, respectively. This thickness was significantly higher than that of other groups, followed by the birds of T3 (1.474 and 0.806 mm) that were significantly higher than those of the T1 birds (control) that were 1.35 and 0.546 mm thickness, respectively.

**Table 7:** Effects of dietary ZONPs supplementation on cell-mediated immune response measured by wattle response to injection of PHA-P of broiler chickens

Groups	Wattle thickness before injection (mm) (M + SE)		Wattle thickness 24 h post-injection (mm) (M + SE)		Mean wattle index (mm) (W.I) (± SE)	Mean relative response (R.R) (± SE)
	R.W.(A)^1^	L.W.(B)^2^	R.W.(A)^1^	L.W.(B)^2^		
T1	1.557 ± 0.05	1.557 ± 0.05	2.103 ± 0.059	1.557 ± 0.05	0.546^c^ ± 0.015	1.351^c^ ± 0.009
T2	1.537 ± 0.064	1.537 ± 0.064	2.443 ± 0.066	1.537 ± 0.064	0.907^a^ ± 0.009	1.590^a^ ± 0.025
T3	1.7 ± 0.088	1.71 ± 0.088	2.52 ± 0.078	1.71 ± 0.088	0.806^b^ ± 0.012	1.474^b^ ± 0.03

Values are expressed as mean ± SEM

Mean wattle index (W.I) = wattle thickness 24 h post-injection – wattle thickness 24 h before injection (for right wattle)

Relative response (R.R) = thickness of wattle after injection/thickness of wattle before injection

1: R.W.(A) = right wattle (injected with PHA)

2: L.W.(B) = left wattle (injected with sterile physiological saline and served as control)

### Intestinal Microbial Count

Table 8 presents the microbial composition in the ileum and cecum of broilers as a result of dietary ZONPs supplementation. The obtained data showed that, as compared with control, both studied doses of ZONPs significantly increased *Lactobacillus* and *Bifidobacterium* counts and significantly decreased *Salmonella* and *E. coli* counts in both ileum and cecum. No significant differences were observed between two doses of ZONPs.

**Table 8:** Effects of dietary ZONPs supplementation on ileum and cecum microbiota of broiler chicken

Groups	Microbial population (Log CFU/g)							
	Cecum				Illeum			
	*Lactobacillus*	*Bifidobacterium*	*E. coli*	*Salmonella*	*Lactobacillus*	*Bifidobacteria*	*E. coli*	*Salmonella*
T1	4.722^b^ ± 0.072	4.422^b^ ± 0.02	4.205^a^ ± 0.126	3.603^a^ ± 0.109	4.248^b^ ± 0.263	4.018^b^ ± 0.147	3.615^a^ ± 0.147	3.133^a^ ± 0.120
T2	5.344^a^ ± 0.089	6.29^a^ ± 0.051	3.316^b^ ± 0.056	3.008^b^ ± 0.055	5.293^a^ ± 0.020	4.940^a^ ± 0.139	2.767^b^ ± 0.118	2.514^b^ ± 0.173
T3	5.424^a^ ± 0.101	6.39^a^ ± 0.036	3.158^b^ ± 0.068	3.030^b^ ± 0.043	5.220^a^ ± 0.091	5.077^a^ ± 0.138	2.792^b^ ± 0.176	2.502^b^ ± 0.257

Values are means ± SEM

^a,b^The same column with different superscripts are significantly different (*P* < 0.05)

### Histological Section

**Fig. 1:**
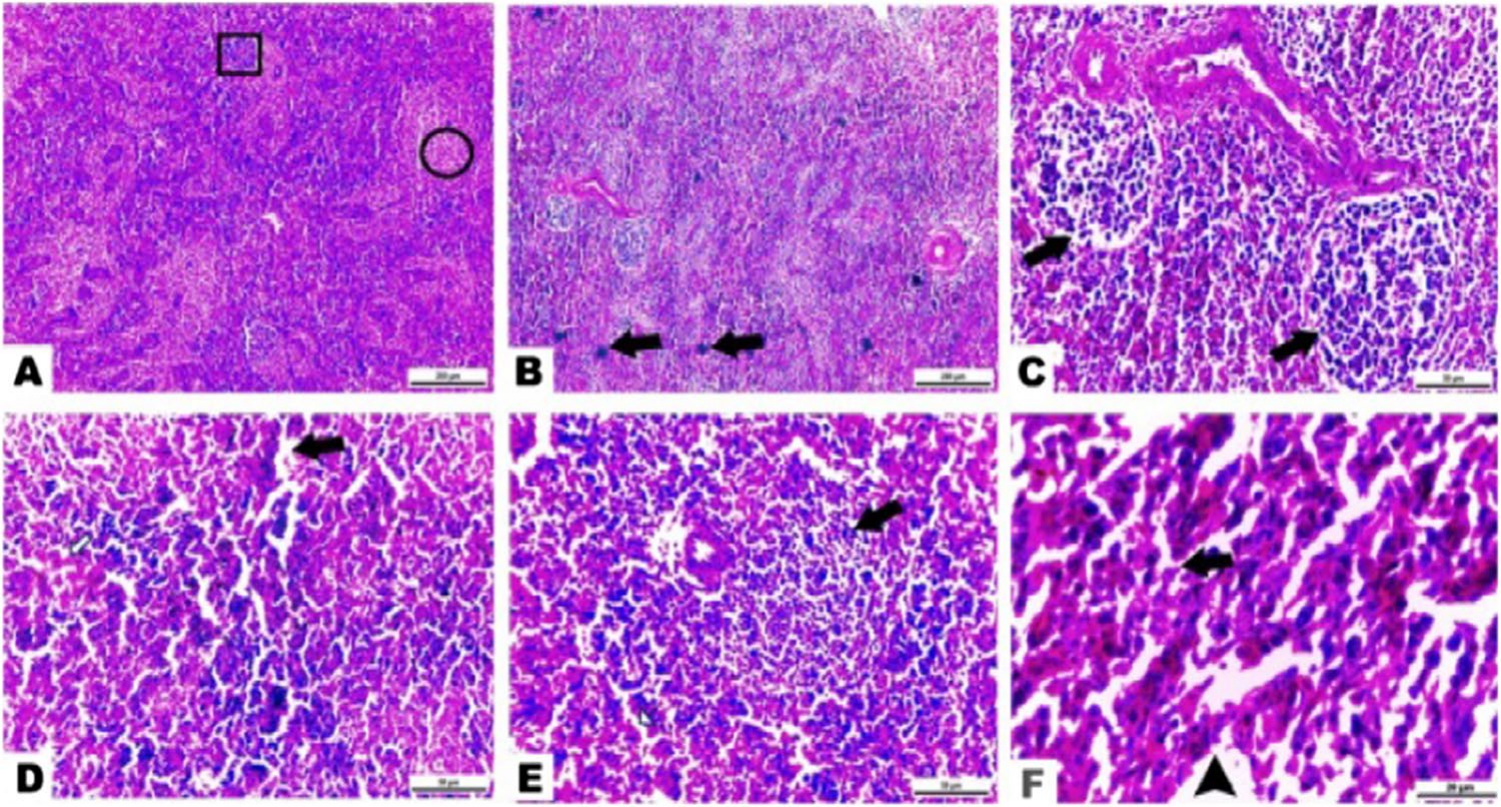
Photomicrographs exhibiting the outcome of ZONPs supplement on the normal splenic architecture of the tested broilers. **A** In the control group, the spleen appeared with a normal structure of white pulp (cube) and red pulp areas (circle). In the 40-mg ZONPs group, **B** extensive mitotic cells (arrows) presented along splenic tissue. **C** Depletion of lymphocytic cells and increase blood cells inside lymph nodules (arrows). **D** Focal necrotic areas (arrows) with inflammatory cell infiltration in a disorganized red pulp structure. In the 60-mg ZONPs group, **E** obvious indistinct white pulp from red pulp area. **F** Red pulp marked a severe necrosis and apoptosis in splenic cords (arrow) with a dilated blood sinusoid (arrowhead) (hematoxylin and eosin stain, magnification power =  × 100, × 400, × 1000; scale bar = 200 µm, 50 µm, 20 µm)

**Fig. 2:**
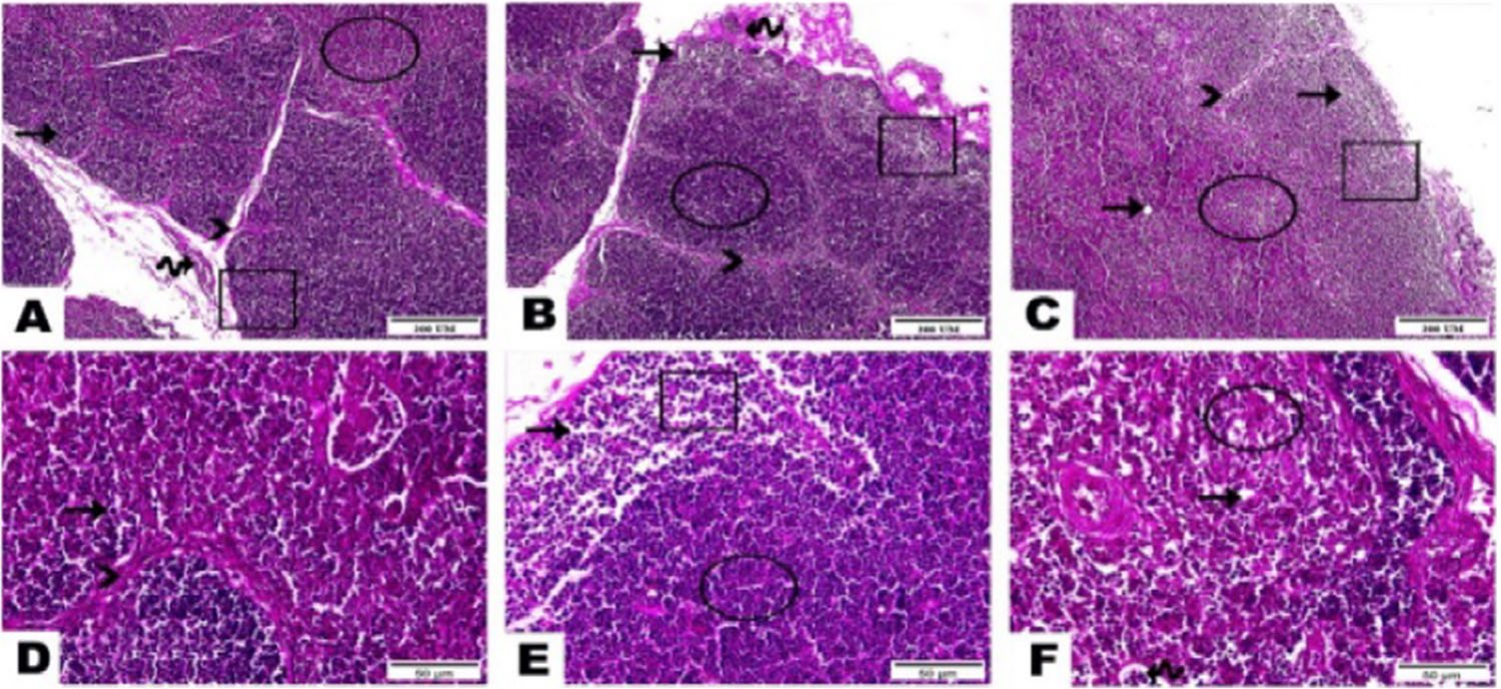
Photomicrographs displayed the histopathological alterations in the thymus of tested broilers as follows: **A** and **D** sections of the control group displayed the normal architecture of the thymus gland as a compact organ with incomplete lobules, each lobule has dark stained outer cortex (cube) and pale stained inner medulla (circle) homing immunocompetent cells mainly, T lymphocyte (arrow). Thymic lobules supported by fine connective tissue septa (arrowhead) and blood vessels (wave arrow). **B** and **E** Sections of the 40-mg ZONPs supplemented group highlighted the faint staining of the thymic cortex along with vacuolations (arrow) and reduction in lymphocyte number (cube). Thymic medulla revealed congestion in between aggregated lymphocytes (circle). Thymic capsule marked with fibrous tissue thickening, congested blood vessels, and infiltration of inflammatory cells (wave arrow). Seta between thymic lobules emphasized a thinning assembly (arrowhead). **C** and **F** Sections of the 60-mg ZONPs supplemented group revealed a very thin fibrous connective tissue septa rendering loss of demarcation between lobules (arrowhead), vacuolations (arrow) caused a light staining of both cortex and medulla (cube and circle, respectively). Notice congested blood vessel (wave arrow) (hematoxylin and eosin stain, photos of **A**, **B**, and **C**: magnification power =  × 100 and scale bar = 200 µm, photos of **E**, **F**, and **G**: magnification power =  × 400 and scale bar = 50 µm)

**Fig. 3:**
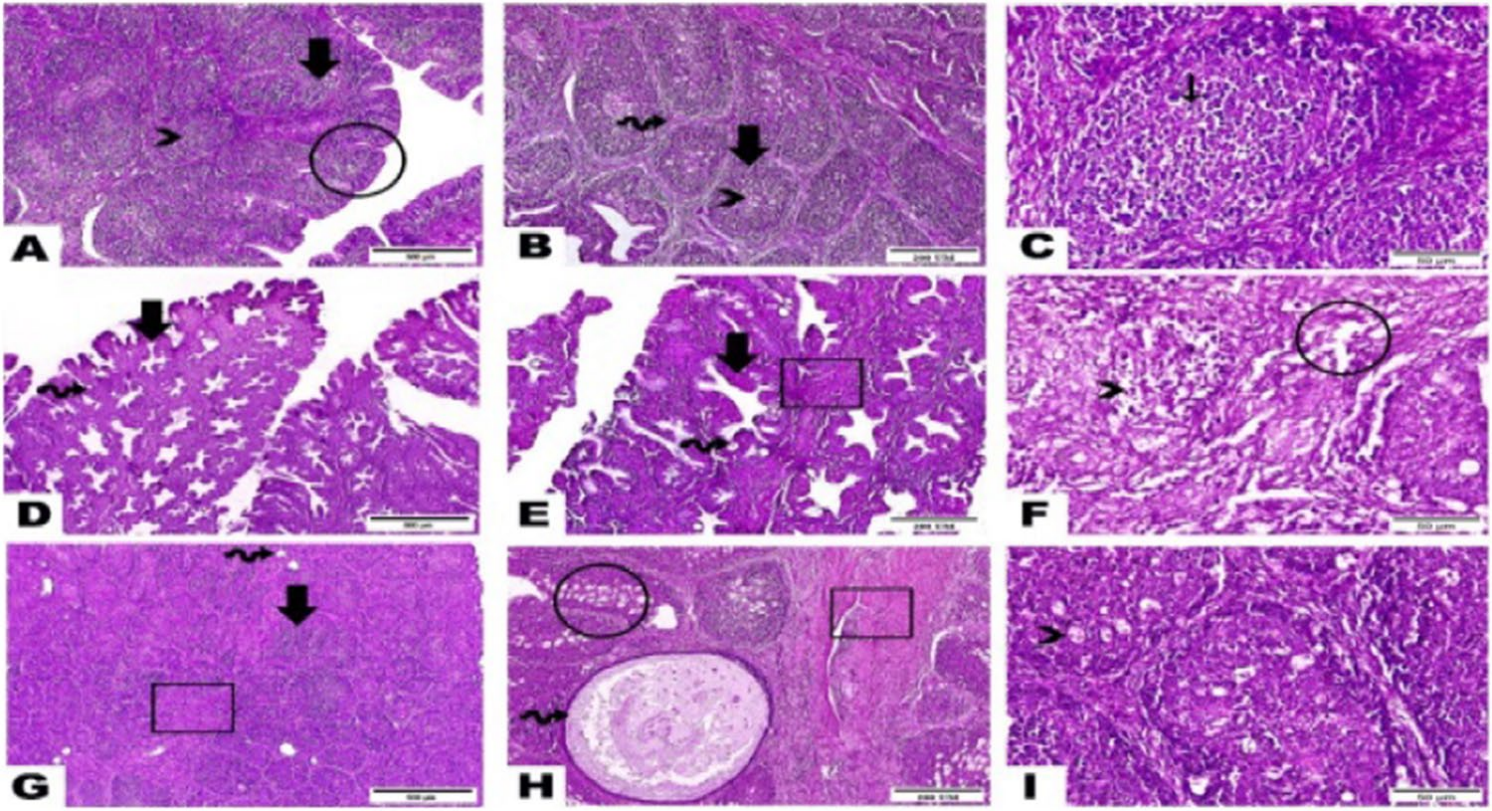
Photomicrographs presented the histopathological modifications in the bursa of tested broilers as follows: **A**, **B**, and **C** Sections of the negative control group showed the normal construction of bursal follicles as an outer cortex (thick arrow), inner medulla (arrowhead), and interfollicular epithelium (circle). Bursal follicles are separated by fibrous connective tissue septa (wave arrow). Notice lymphocytes and plasma cells homing bursal follicle (thin arrow). **D**, **E**, and **F** Sections of the 40-mg ZONPs supplemented group highlighted obvious hyperplasia in the inter-follicular epithelium (arrow), vacuolations (circle), marked thickness in the underlying connective tissue (cube), appearance of multiple epithelial cyst (wave arrow), and decline in lymphocytes number (arrowhead). **G**, **H**, and **I** Sections of the 60-mg ZONPs supplemented group displayed a noticeable increase in the fibrous connective tissue thickness (cube) in between bursal follicles (arrow), high vacuolation within bursal follicle (circle), scattered small, and huge epithelial cyst (wave arrow), along with congested blood vessels (arrowhead) (hematoxylin and eosin stain, photos of **A**, **D**, and **G**: magnification power =  × 40 and scale bar = 500 µm, photos of **B**, **E**, and **H**: magnification power =  × 100 and scale bar = 200 µm, photos of **C**, **F** and **I**: magnification power =  × 400 and scale bar = 50 µm)

Prominent and significant histological changes related to dietary treatments employed in the present study were demonstrated in the spleen, thymus, and bursal tissues (Figs. 1, 2 and 3).

## Discussions

### Relative Organ Weights

**Fig. 4:**
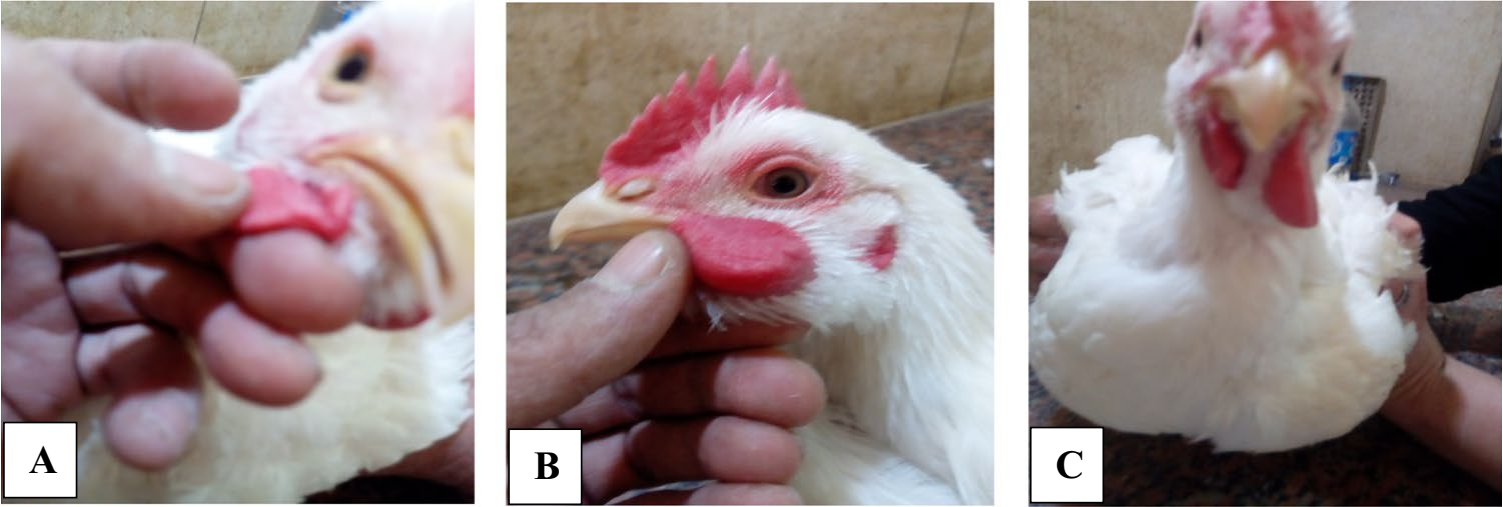
Photomicrograph of left and right wattle as an indicator for cell-mediated immune response after injection with PHA-P. Photomicrograph (**A**) showed a left wattle (served as control) after being injected with 0.1 mL of sterile saline solution. Photomicrograph (**B**) showed a right wattle after being injected with 0.1 mL of PHA-P. Photomicrograph (**C**) showed right and left wattles in broilers after injection

According to the current results and as compared to control, dietary supplementation with ZONPs to broiler chicks decreased the relative kidney, gizzard, and intestine weights and increased the relative spleen, bursa, thymus, and liver weights at both examined doses of ZONPs (Fig. 4). In agreement with our finding, Dosoky et al. [33] reported that relative gizzard and intestinal weights of broilers decreased in response to dietary ZONPs supplementation at dosages of 5, 10, 20, 40, 60, and 80 mg/kg feed. Additionally, when broiler chicks were fed diets supplemented with ZONPs at 40 and 60 ppm, the relative weight of the kidney, gizzard, and intestine was significantly lowered while the liver weight was significantly higher [12]. Furthermore, Reda et al. [7] observed that lowering the ZONPs levels in comparison to other levels resulted in a decrease in the relative weight of the intestine. The decrease in the relative small intestine weight may be explained by enhanced gut endogenous enzyme excretion and activity under adequate Zn supply because Zn is a cofactor for several metalloenzymes associated with better production of digestive enzymes [34]. Moreover, zinc has positive effects on the broiler intestine by improving intestinal integrity, enhancing crypt cell function, barrier structures, and protein synthesis [12]. Increasing liver weight may be attributed to the higher bioavailability of zinc in the form of nanoparticles, which led to higher Zn retention in the liver after absorption [35].

The results of this study pertaining to the relative weight of immune organs supported the better humoral and cell-mediated immune response obtained in the current study (Tables 6 and 7) and confirmed the improved effects of ZONPs on immune organ weights. In line with these findings, Mahmoud et al. [36] found that the relative weights of the spleen and bursa were significantly greater in birds fed diets supplemented with 10, 20, 30, or 40 ppm ZONPs than in the untreated birds. This improvement may be explained by the fact that included nanozinc in diets increased thymulin activity, which boosted T-lymphocyte maturation and stimulated B lymphocyte activation by T-helper cells, improving the immune response [37].

### Hematological Parameters

The present study findings revealed that dietary supplementation with ZONPs caused a significant increase in hemoglobin (Hb%), packed cell volume (PCV%), and red and white blood cell counts (RBCs, WBCs) as compared to the control birds. These results received support from the recent work by El-Maddawy et al. [38] who reported that ZONPs supplementation into Ross broilers diet at 20 ppm significantly increased PCV%, Hb%, and RBCs count. Also, ZONPs supplementation at doses of 5–80 ppm/kg diet had a substantial impact on WBCs, RBC counts, and PCV% [33]. In this respect, Indian River (IR) broilers fed for 5 weeks experienced a significant rise in Hb content and RBCs count when fed a diet supplemented with ZONPs at 3.0 cm^3^/kg feed [14]. In addition, supplementation of zinc nanoparticles at 30–60 mg/kg into broiler diets instead of inorganic source increased WBCs, RBCs counts, Hb%, and PCV% [37]. Due to zinc’s role in erythropoiesis, zinc has long been thought to be involved in the production of RBCs and hemoglobin [39]. Zinc plays a catalytic role in the activity of alfa-aminolaevulinic acid dehydrogenase which is responsible for hem synthesis [40].

### Serum Biochemical Assays

Serum biochemical estimates in the present study as affected by ZONPs supplementation varied significantly in terms of total proteins (TP), globulins (GLOB), AST, ALT ALP, uric acid, and creatinine. Similar results to the present results were reported by Hatab et al. [12] who reported that the highest concentration of serum activity of ALT and AST and lowest concentration of uric acid and creatinine were observed in ZONPs-treated broilers as compared with control birds and altered significantly with increase the inclusion level. Moreover, broilers treated with 40–80 mg/kg of ZONPs had significantly higher serum levels of AST and ALP [33]. In a deep consistent with the present study, Alian and colleagues explained that 40 mg Zn/kg diet was safe in terms of liver and kidney function, as demonstrated by unaltered values of ALT, AST, creatinine, and uric acid in the serum of treated birds compared to control [41]. Furthermore, supplementation of broiler diet with ZONPs at a rate of 50 mg/kg had no impact on the serum levels of AST and ALT [42]. Therefore, serum AST and ALT activities were not significantly altered by the addition of 10, 20, and 40 mg ZONPs/kg diet [43].

Measurements of serum uric acid and creatinine concentrations are the most sensitive indicators to estimate kidney state and functions. Present results revealed that adding ZONPs to broiler diets resulted in significant dose-dependent reductions in serum uric acid and creatinine concentration (Table 4), which was accompanied by a significant decrease in the relative kidney weight (Table 2), reflecting no occurrence of stress or toxicity on kidneys. These results are in a harmony with the recent results by [34, 36]. According to Abdel-Wareth et al. [44], serum uric acid and creatinine levels linearly and quadratically decreased in zinc nanoparticle-supplemented birds at 20, 40, and 60 mg/kg diet**.**

The current results of ALP are in accordance with those published by Samy et al. [6] who found that broilers fed a diet supplemented with green synthesized nano Zn as opposed to traditional form had higher ALP activity. Furthermore, serum ALP activity was significantly increased in Arbor Acers broiler fed on Zn-supplemented ration with 40 and 80 ppm [45]. Due to the fact that Zn is a crucial component of ALP, ALP activity will be employed as one of the indicators to determine the Zn status [46].

With regard to serum proteins, a similar increase in blood proteins and globulins as in the present study was reported when ZONPs were supplemented in broiler diets at 30 and 40 ppm [36] and 20–100 ppm [34]. This increase could be linked to Zn’s essential function in the synthesis of enzymes involved in the production of proteins and nucleic acids. Furthermore, the increased plasma protein concentration in response to zinc treatments may also be attributed to the hormonal regulation of protein metabolism, such as the growth hormone-induced increase in cellular protein production [47].

### Serum Antioxidant

The antioxidant efficacy of ZONPs in the present study was denoted by the significant increase in SOD, GPx, and TAC with a significant decrease in the MDA content in broiler serum and the highest and lowest values appeared in the 40-mg group than in the other treatment groups. Supporting results to the present results were reported by Hassan et al. [48] who found that nano-Zn synthesized by different plant extracts at a level of 70 ppm improved the antioxidant capacity in chickens. Similar results to that obtained in this investigation were also documented by several authors, where supplemented broiler diets with ZONPs at 40 to 80 mg/kg significantly augmented their antioxidant status as shown by elevated serum SOD, GPx, and TAC correlated with decreased MDA concentration [49–51].

The appropriate concentrations of nano-ZnO would stimulate SOD activity and an increase SOD will eliminate ROS production and other processes related to oxidation and eventually lower the MDA concentration [52, 53]. SOD is the major antioxidant enzyme in cellular free radical scavenging processes, and Zn is an essential and main fundamental component in SOD, accounting for about 90% of its structure. Many theories have been proposed to explain the mechanism by which zinc performs its antioxidant action. It has been demonstrated that the presence of zinc in the body causes the production of the free radical scavenger protein called metallothionein, which is rich in cystine and shields cells against lipid peroxidation and free radicals [43].

Our findings suggest that the use of nano-sized zinc oxide might strengthen the oxidative defenses by increasing SOD, GPx, and TAC and decreasing MDA concentration, which would contribute to improve the immunological response as previously shown in Tables 6 and 7. The enzyme SOD exerts an important function in preserving the redox balance of the bird’s immune system by eliminating reactive oxygen species [54].

### Immunological Responses

Results obtained in the present study indicated that ZONPs supplementation at levels examined in broiler diets improves both types of immune responses, humoral and cellular, at all test periods as compared to the control group**.** One of the most profound effects of ZONPs supplementation is its ability to improve immune system functions. The positive effect of ZONPs on immune response through the current study is corroborated with results by Zarghi et al. [55] who reported that the humoral immune response measured by antibody titer to SRBCs inoculation and cellular immune responses measured by cutaneous basophil hypersensitivity (CBH) elicited by Phytohemagglutinin-P (PHA-P) injection were increased significantly in Ross broilers supplemented with zinc in their diets. Furthermore, the authors of Dosoky et al. [33] and Zhang et al. [49] observed improved cellular immunity in chicks fed a diet supplemented with ZONPs as evidenced by notable variations in the phagocytic activity, phagocytic index, IgM, and IgG.

Similar to the present study, a significant increase in antibody titer against a specific antigen (SRBC/NDV virus) was previously reported in the literature and has been attributed to Zn supplementation in broilers [56, 57]. In this respect, the findings of Sagar et al. (**58**) and Sunder et al. (**18**) also provided additional support to the current findings, and they stated that broilers fed diets supplemented with 40 ppm Zn had significantly heavier spleen and bursa weights and increased wattle thickness when compared to controls, suggesting that 40 ppm of zinc was sufficient to lymphocyte development and triggering immune response.

The significance of zinc in boosting immunity has been studied in a number of research and has suggested various mechanisms for this effect. It has been established that Zn is a crucial co-factor that influences the production and activity of thymulin (a thymic hormone that contains Zn) from the thymus gland. Thymulin binds to the surface receptors of T lymphocytes to stimulate and control the maturation and activation of these cells, which in turn promotes the development and differentiation of B lymphocytes by T-helper cells, which in turn triggers the release of macrophages, thereby enhancing the immune response [58]. Besides, Zn’s antibacterial properties, which reduce the pathogenic microbial load and boost gut health, may account for the improvement in immune response following the dietary addition of Zn [59]. Our results (Table 8) imply that the use of nanoscale zinc oxide will result in a reduction in harmful bacteria and an increase in beneficial bacteria which would boost the immune response (Tables 6 and 7).

### Intestinal Microbial Counts

The current study’s findings showed that dietary ZONPs supplementation at 40 and 60 ppm to broiler chickens increased the population of beneficial microorganisms (*Bifidobacterium* spp. and *Lactobacillus* spp.) and decreased the undesired microorganism (*E. coli* and *Salmonella* spp.) in both the ileum and cecum. Previous reports indicated similar potential effects of ZONPs to modify and fortify the composition of the intestinal microbiota of broilers**.** Reda et al. [7] reported a decrease in the number of *E. coli*, *Salmonella*, and *Enterococcus* spp. and an increase in the beneficial microbial community in the cecum as a result of dietary supplementation with biological ZONPs at levels of 0.1 and 0.3 g/kg diet. This beneficial effect of zinc could be attributed to its role in the regulation of cecal microbial community in broilers by decreasing the number of *Salmonella* and increasing the number of useful *Lactobacillus* bacteria [60].

The diverse antibacterial property of nanomaterials is mostly and inversely correlated with particle size, meaning that the smaller the particle, the higher the specific surface area to volume ratio, which improve their availability and accessibility for interacting with bacteria [8].

The antimicrobial mechanisms of ZONPs are through an electrostatic attachment between the NPs and the bacterial cell membrane because microorganisms have a negative charge and metal oxides have a positive charge; this interaction leads to bacterial cell membrane integrity being impairment and deformation, which leads to loss of membrane permeability that influence the transport throughout the cell membrane and then ZONPs permeating the cell membrane and penetrating into the cells**.** When ZONPs penetrate and enter bacteria, they interact with phosphorus and sulfur-containing compounds like the DNA of bacteria to provide bactericidal activity [61, 62]. Additionally, ZONPs cause the generation of reactive oxygen species, such as H_2_O_2_, a potent oxidizing agent that puts cells under oxidative stress, disrupting metabolic processes and damaging cellular components like lipids and proteins, leading to cell death. The formation of ROS is a common antibacterial activity strategy adopted by ZONPs [62, 63].

The intrinsic and inherent properties of ZONPs in preventing the growth of a wide spectrum of pathogens and the nonspecific mode of action against bacteria make them excellent candidates as antimicrobial agents replacement for conventional antibiotics without risk of developing bacterial resistance [64, 65].

## Conclusion

Under the condition of this study and from the obtained results, a conclusion could be drawn that dietary supplementation of biogenic synthesized ZONPs at an inclusion level of 40 or 60 mg/kg diet had significant beneficial effects on blood indices, physiological, antioxidant status, immunological response and modifying the intestinal microbial population and finally health status of broiler chicks. Current results also showed that the delivery of Zn in the form of nanoparticles at a concentration of 40 mg/kg to broiler diets was more efficient and could be used as a safe dose without any detrimental effects than high levels.

## Data Availability

All data generated or analyzed during this study are included in this published article.
